# Isolation of a *Stenotrophomonas* strain and identification of methyltransferase genes conferring the high arsenic volatilizing ability

**DOI:** 10.1128/aem.02467-24

**Published:** 2025-05-30

**Authors:** Diksha Singh, Nitish Sharma, Sheetal Agarwal, Sadaf Aiman Khan, Veena Jain, Sukhveer Singh, Somendu Roy, Kusum Yadav, Sudhir Pratap Singh, Vikas Srivastava

**Affiliations:** 1 Systems Toxicology Group, FEST Division, CSIR - Indian Institute of Toxicology Research 538266, Lucknow, Uttar Pradesh, India; 2 Department of Biochemistry, University of Lucknow, Lucknow, Uttar Pradesh, India; 3 Center of Innovative and Applied Bioprocessing (DBT-CIAB) Mohali 508486 , Sahibzada Ajit Singh Nagar, Punjab, India; 4 Department of Biological Sciences, Tokyo Metropolitan University 12944, Tokyo, Japan; 5 The University of Queensland - Indian Institute of Technology Delhi Academy of Research (UQIDAR), Indian Institute of Technology Delhi 28817, New Delhi, India; 6 Academy of Scientific and Innovative Research (AcSIR) 550336, Ghaziabad, Uttar Pradesh, India; 7 Department of Industrial Biotechnology, Gujarat Biotechnology University 633303, Gandhinagar, Gujarat, India; Colorado School of Mines, Golden, Colorado, USA

**Keywords:** arsenic, bioremediation, *Stenotrophomonas*, novel strain, arsenic methyltransferase, arsenic volatilization, ArsM, gene expression, gene transfer, genome sequencing

## Abstract

Microbial arsenic (As) volatilization plays a significant role in the global As biogeochemical cycle and presents a promising approach for bioremediation. In this study, an autotrophic strain of *Stenotrophomonas maltophilia* was isolated from sewage water containing 75 ppb of arsenic, exhibiting a high arsenic metabolism and volatilization rate. This strain tolerated arsenic concentrations ranging from 10 to 500 ppm. Volatilization tests were conducted at an environmental median of 200 ppb (0.2 ppm) As. The strain eliminated 50% of the total arsenic within 48 h, and 0.1 ppm (101 ppb) of arsenic was reported to be trapped in the headspace of the culture tube, confirming its volatilization. After genome isolation, sequencing, and assembly, the existence of the As metabolizing operon (*ars*RABC), as well as other As-resistance conferring genes (*ars*M, *ars*I, *aio*A, *aox*B, and *arx*A), was identified using different annotation methodologies. The genes conferring resistance against different heavy metals and multiple drugs used as antimicrobials were also identified in the genome assembly of the isolate. Based on the gene expression measured using real-time polymerase chain reaction, complete gene sequences of four *ars*M genes were mined, cloned, and expressed in *Escherichia coli* BL21(DE3) cells. The cells harboring recombinant plasmid of each construct could volatilize As up to 86 ppb after 48 h incubation in arsenic-enriched media. The isolated strain of *Stenotrophomonas* and its heterologously expressed methyltransferase genes could be a potent tool for permanently removing arsenic from water samples by volatilization.

**IMPORTANCE:**

Arsenic contamination in water, soil, and air poses significant health and environmental risks, as inorganic arsenic compounds are highly toxic and carcinogenic. Microorganisms capable of transforming arsenic into volatile forms play a pivotal role in the biogeochemical cycling of this metalloid, reducing its bioavailability and toxicity in contaminated environments. In this work, a strain of *Stenotrophomonas* sp. was isolated from the sewage water and tested for its ability to survive in minimal arsenic media. The strain was found to be highly resistant to arsenic and volatilized more than 50% of the arsenic from the growth media. The putative methyltransferase genes from the isolated strain, when heterologously expressed in *Escherichia coli*, conferred an ability to volatilize arsenic in the recombinant host, too. Therefore, the isolated microorganism offers a natural, eco-friendly alternative to conventional chemical methods, making it an important tool for addressing arsenic biosafety issues in the environment.

## INTRODUCTION

Arsenic (As) is the 20th most abundant element in the Earth’s crust, occurring naturally and widely dispersed with an average concentration of 1–2 mg/kg (1). It infiltrates from aquifers through rainwater leaching, weathering, seismic and volcanic activities, etc. Another source of As includes fumes and wastes transported by natural vectors like wind and water (2). Millions of people get affected by As exposure through food, air, water, and soil. In nature, As is found in several oxidation states, including arsenite (As^3+^), elemental arsenic (As^0^), arsenide (As^3−^), and arsenate (As^5+^). According to the United States Environmental Protection Agency (USEPA) and the Agency for Dangerous Substances and Disease Registry (ATSDR), it is a hazardous compound ranked first on their priority list of dangerous substances. Acute exposure to As could result in vomiting, abdominal pain, and diarrhea, followed by numbness and tingling of the extremities, muscle cramping, and even death in extreme cases. Long-term exposure to inorganic As causes changes in pigmentation, skin lesions, hard patches on the palms and soles of the feet called hyperkeratosis, and skin cancer (3). As is also associated with adverse pregnancy outcomes, such as infant mortality. *In utero* exposure is linked to mortality in young adults due to multiple reasons like cancer, lung diseases, cardiac arrest, and kidney failure (4). However, As has also been used as a medicine in the treatment of diseases like psoriasis and syphilis (5), hematological malignancies, and cancer (6). The water contains both the trivalent arsenite (As^3+^) and the pentavalent arsenate (As^5+^) forms of As (7). An estimated 140 million people across 50 nations have been reported to be impacted by contaminated drinking water, which has more As than the WHO’s standard. The high concentration of inorganic As has been documented in several nations’ groundwater, including Argentina, Bangladesh, Chile, China, India, Mexico, the United States of America, etc. (8, 9).

Removal of As from groundwater poses severe ordeals due to its high solubility. Some traditional methods, such as coagulation, membrane filtration, reverse osmosis, adsorption, and filtration, are used for its removal (10). However, in all aforesaid methods, the change in the oxidation state of As from highly toxic arsenite to somewhat mild arsenate is done either by reaction of oxygen with As under normal atmospheric conditions or by chemical oxidants like hydrogen peroxide, chlorine, and ozone. These chemical oxidants are costly and harmful, which signifies the importance of finding sustainable methods to remove As from groundwater (11). Various physical and chemical methods are available for As removal from the soil, and the physical approach involves reducing its concentration by blending contaminated and uncontaminated soils, effectively diluting the contamination (12). Another method, “soil washing,” involves using several chemicals, including hydrogen bromide, phosphoric acid, nitric acid, and sulfuric acid, to wash the contaminated soil. However, the biosafety aspect of chemicals and their cost ineffectiveness prevent soil washing from being a widely used method, limiting its application to a smaller-scale enterprise (13). Cement has also successfully stabilized As-rich sludges, especially to immobilize the soluble arsenite. However, when stored longer, this method does not meet the regulatory criteria for As leachability. Contemporary chemical remediation methods primarily encompass techniques, such as employing specific media for adsorption, immobilization, modifying coagulation in conjunction with filtration, precipitation, and engaging in complexation reactions (14). The coagulation with filtration approach is a financially sustainable way to remove As from polluted sources. However, its efficiency is usually lower (<90%). The drawbacks of physical and chemical approaches for As removal lie in their low cost-effectiveness and limited scalability, making them more suitable for smaller-scale applications. Consequently, there has been a shift towards embracing phytoremediation and biological remediation of As to address these limitations.

Microorganisms can interact with As through various mechanisms, such as absorption, mobilization, precipitation, redox reaction, and methylation (15). Microbes are exposed to metallic ions in their surroundings; some of these ions, such as magnesium, potassium, copper, and zinc, are absorbed as nutrients, while other ions, such as mercury, lead, cadmium, and As, are poisonous and are eliminated from the microbial cells (16). Bacteria uptake arsenite and arsenate using glycerol and phosphate transporters because of their chemical similarities to arsenite and arsenate (17). Many Gram-positive and Gram-negative bacteria use As-resistant systems to detoxify cells (18) and reduce As concentration in the environment. Bacteria also employ a variety of reduction and oxidation processes for this detoxification process (19). Previous reports showing As removal by microorganisms emphasize either the transformation of As from its inorganic to organic form or simple extermination using efflux pumps. A study by Altowayti et al. (20) reported that *Bacillus thuringiensis* strain WS3, *Pseudomonas stutzeri* strain WS9, and *Micrococcus yunnanensis* strain WS11 remove arsenite and arsenate via bio-adsorption up to 98% and 95%, respectively (20). Also, Zacarías-Estrada demonstrated sulfate-reducing bacteria removing 98% As in anaerobic sludge enriched with As (21). Another study by Razzak et al. has demonstrated a model to remove arsenite and iron with the help of coconut husk using Fe-oxidizing bacteria, e.g., *Leptothrix* spp. (22). In a study, Huang isolated a novel bacterium strain, SM-1, from the family Cytophagaceae, representing a new species and genus. The introduction of strain SM-1 significantly enhanced As methylation and volatilization in the soil. In a study, the arsenite methyltransferase gene (*ars*M) was cloned from SM-1, and when expressed in *Escherichia coli*, it conferred the ability to methylate and volatilize As (23–25).

Microorganisms have evolved genetic systems, such as the *Ars* operon, to cope with As toxicity due to prolonged exposure in As-contaminated environments. The Ars operon is commonly found in prokaryotes and is often more prevalent than genes involved in other essential metabolic processes, such as tryptophan synthesis (26). The *Ars* operon typically consists of three core genes: *ars*R, *ars*B, and *ars*C. These genes encode a transcriptional repressor (ArsR), a transmembrane efflux pump (ArsB), and an arsenate reductase (ArsC), respectively, which work together to detoxify As by reducing arsenate (As⁵^+^) to arsenite (As³^+^) and expelling it from the cell (27). In addition to the detoxification pathways, some facultative or obligate bacteria utilize As in metabolic processes. These bacteria can reduce arsenate to arsenite using arsenate respiratory reductase (encoded by the *arrAB* operon) during anaerobic respiration (28). Additionally, arsenite can serve as an electron donor under both aerobic and anaerobic conditions. In aerobic conditions, arsenite is oxidized *via* enzymes encoded by the *aioAB* (or *aoxAB*) operon, which converts arsenite (As³^+^) into the less toxic arsenate (As⁵^+^). Under anaerobic conditions, a different arsenite oxidase, encoded by the *arxA* gene, performs a similar function, enabling As oxidation without oxygen (29).

This study isolated an autotrophic strain of *Stenotrophomonas maltophilia* from sewage water containing approximately 75 ppb of As. This strain demonstrated a remarkable capacity for As volatilization, enabling it to tolerate high levels of As. To explore its potential for practical applications, we tested the strain’s ability to remove As from tap water artificially spiked with As. The results confirmed that the isolated *S. maltophilia* could effectively remove As from water, even without organic carbon sources, supporting its potential for groundwater remediation in natural and laboratory conditions. Genome sequencing of the isolated strain identified As volatilization genes and also genes associated with heavy metal and multidrug resistance, highlighting its usefulness in As detoxification through genetic manipulation of a non-pathogenic host. However, being an opportunistic pathogen, the new *S. maltophilia* strain cannot be used directly for As bioremediation in drinking water systems due to safety concerns. Therefore, to address this, the As methylation genes from *S. maltophilia* were successfully cloned and expressed in a non-pathogenic strain of *E. coli*, providing a safer alternative for As removal and bioremediation.

## MATERIALS AND METHODS

### Materials

Sodium meta arsenite (cat no. S7400-100G), kanamycin (cat no. 6061-5G), phenol: chloroform: isoamyl alcohol (PCI) (cat no. P2069-400 ml), hydrochloric acid (HCL), nitric acid (HNO_3_), and isopropyl β-D-1-thiogalactopyranoside (IPTG) were purchased from Sigma, India. Agarose was purchased from Lonza, India (cat no. 50004-500G). Potassium iodide (cat no. 78240) and ascorbic acid (cat no. 65265) was purchased from SRL Diagnostics, India. Minimal media (M9) (cat no. G013-500G), Luria Broth (LB) media (cat no. M575-500G), and Agar type 1 (cat no. GRM666-500G) were purchased from Himedia, India. Emerald master mix (cat no. RR320) and TB Green Premix Ex Taq (cat no. RR820A) for PCR and qPCR amplification were purchased from Takara, Japan. Trizol reagent (cat no: 15596026) for RNA isolation was purchased from Thermo Fisher Scientific, India. DNA purification kit for gels (cat no. 1501004) was purchased from Qiagen, India. All the primers used in this study were designed with the help of Primer3 web software (https://primer3.ut.ee/), and their sequences and other details have been given in Tables 1 and 2.

**TABLE 1:** Primers used for screening of arsenic metabolizing gene by PCR amplification

Gene	Forward primer	Reverse primer	Amplicon length	Reference
16srRNA 1	5′CCAGCAGCCGCGGTAATACG3′	5′ATCGGCTACCTTGTTACGACTTC3′	1,300 bp	
16srRNA 2	5′AGAGTTTGATCCTGGCTCAG3′	5′GGTTACCTTGTTACGACTT3′	996 bp	
arsC	5′GCTACGTCTCTCTCTGTCACATTGTA3′	5′CTGCTTCATCAACGACTTTTTC3′	409 bp	(30)
a**r**sB	5′CCCTGTCAGGAGGTTTTATGTTA3′	5′GCAGGCTGGGTTATGATAAATAG3′	1,300 bp	(30)
aioA	5′TGCCGAACTTGTGGGTGTAG3′	5′CCAAGGAGGCGGAGAAGTTT3′	1,114 bp	(31)
arxA	5′GGACTAGTAGACCCGTGTGACCAAGA3′	5′GGACTAGTTGGTGTTGCGGTAGTCGTA3′	250–300 bp	(32)
aoxB	5′GTGGTCTTGTAGTGGTCGCA3′	5′CGTCATGTTCGCCTACGAGA3′	250–300 bp	(33)
arrA	5′CACAGCGCCATCTGCGCCGA3′	5′CCGACGAACTCCTTGTTCCA3′	300–350 bp	(32)
arsR	5′ATCAGGAGCGCCATATGT3′	5′TCCCGGATAAAACACATCTG3′	699 bp	(34)
arsM	5′TCTCTCGGCTGCGGCAATCCCAC3′	5′CGACCGCCAGGCTTCAGTACCCG3′	1,000 bp	(35)
arsI	5′GGAGGGAGAATTCATGAAATATGCGC3′	5′TGTGTAAGCTTTTATTCAACAGTTGTC3′	250–500 bp	(36)

**TABLE 2:** Primer details of isolated sequence

Sequence name	Forward primer	Reverse primer	Product size
Sequence 1	TGCTGGATATCGCCTCTGG	GATCGAAGCTGCTGTCCTTG	189
Sequence 6	GATGGCCAGCTTCTACAACC	GCTGCCAGTCTTCCATGAAC	245

### Collection of water samples

Water samples were collected aseptically from 10 distinct regions of Lucknow, which included areas affected by hospital, industrial, and domestic waste efflux.

Samples were collected in sterile bottles, kept in a 4°C storage box, and shipped to the laboratory at the Indian Institute of Toxicology Research (IITR), a national institution of the Council of Scientific and Industrial Research (CSIR) Lucknow, India, and stored at 4°C for further processing. The samples were separated in aliquots for additional experiments like elemental analysis, microbial isolation, and other estimations.

### Heavy metal analysis

The concentrations of heavy metals in the water samples were determined using inductively coupled plasma mass spectrometry (ICP-MS) by Qtegra Intelligent Scientific Data Solution software by Thermo Fischer Scientific. For the analysis, 1 mL of the sample was passed through a 0.22 µm membrane filter and digested with 1% HNO_3_ at 50°C for 5 min, followed by Thermo Fisher Scientific protocol. The digestion was followed by measuring the concentration of different heavy metals, viz., Arsenic (As), Cobalt (Co), Chromium (Cr), Copper (Cu), Nickel (Ni), Cadmium (Cd), Mercury (Hg), Lead (Pb), etc. present in the samples. The multi-metal standard in different concentrations ranging from 10 to 50 ppb was used to estimate the compositions of metals and metalloids in water samples.

### Screening of As metabolizing genes

Total genomic DNA was extracted from the water samples using the phenol: chloroform: isoamyl alcohol (PCI) extraction method (37). Gene-specific primers specified in Table 1 were used to perform PCR amplification to determine the presence of the As metabolizing genes in the water samples, while 16s rDNA primer was used as an internal control. Amplification was done using nine different sets of primers specific for As-resistance conferring systems (*ars* operon), arsenite oxidation (*aioA, arxA,* and *aoxB*), and As methylation genes. The reaction was performed in 50 µL of reaction mixture that contained 25 µL of Emerald Amp MAX PCR Master mix (2× premix), 100 ng of template DNA extracted from water samples, forward and reverse primer in 0.2 µM concentration, and deionized water for volume makeup. The reaction conditions included initial denaturation at 98°C for 10 s, followed by denaturation, extension at 60°C for 30 s, and amplifications at 72°C for 1 min/kbp for 30 cycles. The presence of As-related genes was determined by running the amplified PCR products on a 1.5% agarose gel.

### Isolation of As-tolerant bacteria

Out of 10 water samples, sample 8 was selected for bacterial isolation due to its high As content and detection of different genes related to As metabolism. The aforesaid water sample was filtered through Whatman filter paper, serially diluted for optimal growth count, and plated on LB agar plates containing kanamycin antibiotics (50 µg/mL concentration) to assess the level of antibiotic resistance and also to reduce the overgrowth of other environmental microbes (33, 38, 39). Following a 30-day incubation of the bacteria with 10 ppm As in an M9 minimal medium, nine distinct colonies, designated C1, C2, C3, C4, C5, C6, C7, C8, and C9, exhibited growth in the presence of As. To assess their survival, these colonies were reinoculated into an M9 minimal medium supplemented with 10 ppm As but without an external carbon source (40). After an additional 30-day incubation period, three colonies, C1, C5, and C8, demonstrated enhanced survival in the presence of As in minimal media. The presence and expression of As-metabolizing genes were verified using an *in silico* approach to investigate As metabolism in these strains further. All experiments were performed in triplicate to ensure reproducibility and to assess biological variability.

### Assessment of microbial survival in the presence of As

Three colonies, marked as C1, C5, and C8, were exposed to sodium meta-arsenite concentrations of 10, 100, 250, and 500 ppm to evaluate their response to the increasing As levels. The colonies were incubated in M9 minimal medium at 37°C with shaking at 150 rpm for 14 days. Cell survival was monitored at various time points by plating aliquots on LB agar plates. Colonies that exhibited resistance to As were subsequently inoculated into a modified M9 minimal medium containing M9 salts (disodium hydrogen phosphate Na_2_HPO_4_, potassium dihydrogen phosphate KH_2_PO_4_, sodium chloride NaCl, and ammonium chloride NH_4_Cl), 1 M CaCl₂, and 1 M MgSO₄, with sodium meta-arsenite but no additional carbon source. The colonies were grown in a medium described above at 37°C and 150 rpm for 14 days. To assess bacterial survival, samples were collected at 48 h and again on day 14. Colony-forming units (CFU/mL) were determined by measuring absorbance at 600 nm (OD_600_) and performing serial dilutions and plating on LB agar. All experiments were conducted in triplicate to ensure biological reproducibility.

### Gene expression analysis

Real-time PCR (RT-PCR) was used to measure the expression of genes that metabolize As in the isolated colonies. Then, 1 mL of bacterial culture was collected as a sample for RNA isolation, and complementary DNA (cDNA) synthesis was done after 48 h and 14 days of incubation. RNA isolation was performed using the TRIzol reagent following the manufacturer’s protocol. The RNA pellet was cleaned and centrifuged at 7,500 × *g* for 10 min at 4°C, then air-dried and resuspended in nuclease-free water. cDNA synthesis was done using a high-capacity cDNA reverse transcription kit, following the standard protocol. RT-PCR expression profiling was conducted using the TB Green Premix Ex Taq (Tli RNaseH Plus) on an ABI QuantStudio 6 Flex RT-PCR System, adhering to the manufacturer’s guidelines. The primers used for gene expression analysis are listed in Table 1, with 16S rRNA employed as an internal control for normalization of results.

### Measurement of As concentration

Atomic fluorescence spectroscopy (AFS) using an atomic fluorescence spectrophotometer AF420 (PG Instrument) was employed to quantify As concentrations in the media and cells. Bacterial cultures (1 mL) were centrifuged at 8,000 × *g* for 10 min at 50°C to pellet the cells. The cell pellet was digested with aqua regia (3:1 HCl₃: HNO_3_), and the supernatant was filtered through a 0.22 µm membrane filter and digested with 1% HNO₃ at 50°C for 10 min. Following digestion, each sample was derivatized with a 1 M potassium iodide and ascorbic acid solution, diluted 1,000× with 10% HCl, and analyzed by AFS. All experiments were conducted in triplicate to ensure biological reproducibility.

### Assessment of As removal

To evaluate As removal by the isolates at an environmentally relevant dose, colonies were exposed to 0.2 ppm As, a concentration commonly reported in As-endemic areas (25). The colonies were cultured in an M9 minimal medium containing 0.2 ppm As for up to 96 h. After 48 and 96 h, 1 mL of the culture was centrifuged at 8,000 × *g* for 10 min to pellet the cells. The supernatant was collected in separate tubes, and the cell pellet was digested with aqua regia (3:1 HCl₃:HNO_3_) at 50°C for 10 min. The supernatant was treated with 1% HNO₃. Following digestion, the samples were diluted, derivatized, and analyzed using atomic fluorescence spectroscopy (AFS). All experiments were conducted in triplicate to ensure biological reproducibility.

### Validation of As volatilization

The As volatilization potential of colonies C1, C5, and C8 was evaluated using a trapping method. Colonies C5 and C8 demonstrated significant volatilization and were selected for further experiments. The colonies were incubated in an M9 minimal medium containing 0.2 ppm As at 37°C with shaking at 150 rpm for 48 h. To capture volatilized As, culture tubes with nitrocellulose membranes were impregnated with 6% hydrogen peroxide (H₂O₂) and sealed following a previously established protocol (26). After incubation, culture media were centrifuged at 8,000 × *g* for 10 min at 27°C. The supernatant was treated with 1% HNO₃ and heated at 50°C for 10 min. To quantify trapped As, the nitrocellulose membrane was digested with concentrated HNO₃ at 50°C for 10 min. All digested samples were analyzed for As content using atomic fluorescence spectroscopy (AFS). All experiments were conducted in triplicate to ensure statistical significance.

### Assessment of As volatilization in a simulated natural environment

To evaluate the As volatilization efficacy of colonies C5 and C8 in a simulated natural environment, the colonies were cultivated in tap water supplemented with 0.2 ppm As. The strain of *E. coli* was used as a control. Each colony was incubated in 5 mL of tap water containing 0.2 ppm As in sealed flasks, covered with aluminium foil, and left at room temperature for 72 h. Samples (1 mL) were collected at 0, 48, and 72 h for further analysis. These samples were centrifuged at 8,000 × *g* for 10 min at 27°C and digested with 1% HNO₃ at 50°C for 10 min before As quantification was done using atomic fluorescence spectroscopy (AFS).

### Identification of methyltransferase gene

The whole genome sequencing of isolated colonies C5 and C8 was performed to analyse their genomic architecture as well as the presence of metal resistance-conferring genes. The isolated genomic DNA was sequenced using Sanger sequencing technology (Eurofins Scientific, India). The quality assessment of generated Fastq reads was performed by trimming the adapter sequences using Trimmomatic (41). Trimmed reads were subjected to the quality check using the PRINSEQ tool (28) to evaluate any adapter contamination, GC content, constant read lengths, and the presence of any ambiguous Ns among the reads. The high-quality reads were assembled into the larger contigs on default parameters using the de Bruijn graph-based Spades tool (42), which is a Quast tool (43) was used for quality check of the assembled genome on default parameters. The integrity, completeness, contamination, and heterogeneity among genome assemblies were checked using the CheckM v1.0.18 tool at default parameters (44). Further, the whole genome taxonomy of the sequenced isolate was established using the tree-based taxonomy tool GTDB-tk v1.7.0 at KBase (45, 46). Different operons and regulating genes among the genomes of colonies were predicted using a web-based tool (47). Circular representations of the genome showing different attributes like open reading frames (ORFs), GC content, genes involved in basic metabolism of defense, virulence, recombination, and repair, etc., were shown using the web-based tool Proksee (48). The genome assembly in FASTA format was used as input, and different operons and genes were predicted using default parameters. The predicted genes were annotated for their functional descriptions using cluster of orthologous genes (COG) assignments. Apart from evaluating the genes involved in basic microbial metabolism, various methyltransferase-encoding genes were identified from the predicted operons in the genome. This information was further validated by extracting the entire ORFs and their corresponding protein coding sequence (CDS) and individually subjecting them to the BLAST analysis against the NCBI non-redundant database. Among various putative methyltransferase encoding genes, some putative variants were further amplified, heterologously expressed, and characterized to test their As volatilization capabilities.

### Heterologous expression of methyltransferase genes

From *in silico* operon mapping analysis of *S. maltophilia* and the real-time PCR data of upregulated transcripts, two gene sequences, *3d1* and *3d6*, encoding for methyltransferases as mentioned in Table 2, were further characterized for their encoded proteins. The genes were amplified from the genomic DNA of the isolate by using gene-specific primer pairs (Table 1). The amplified PCR product was cloned into the pJET 1.2 cloning vector (Thermo Fisher Scientific, India) and sequenced. The genes were cloned into the expression vector pET28a (+) under *Nde*I and *Xho*I restriction sites. The confirmed constructs were transferred to *E. coli* Rosetta(DE3)pLysS cells (Novagen, Inc. United States) for heterologous expression of proteins.

### As volatilization using recombinant *E. coli*

The recombinant *E. coli* cells were revived in LB medium and incubated in a minimal medium containing 0.2 ppm As at 150 rpm and 37°C for 48 h. As-containing media without any microbial inoculum were also included as a control. The protein expression was induced by introducing the IPTG (0.1 mM) after the culture O.D._600_ reached between 0.4–0.5. The procedure for trapping and estimating the volatilized As was followed as described in previous sections. After 48 h of incubation, cells were collected by centrifugation, and the supernatant was transferred to separate tubes for further analysis. The nitrocellulose membrane used for trapping As was digested with concentrated HNO₃ for subsequent atomic fluorescence spectroscopy (AFS) analysis. The supernatant was digested with 1% HNO₃ by heating at 50°C. To determine the concentration of volatilized As trapped in the nitrocellulose membrane, 100 µL of the digested sample was derivatized with ascorbic acid and potassium iodide (KI) (each 10% w/v in 10% HCl). The supernatant was diluted with 10% HCl to ensure the response intensity remained within the linear range. A standard As solution (10 ng/mL) was prepared, and a calibration curve was plotted within the 0.5 to 4 ng/mL range. The limit of detection (LOD) for As was 0.037 ng/mL, and the limit of quantification (LOQ) was 0.10142 ng/mL. The concentration of As in each sample was calculated based on the calibration curve. All experiments were conducted in triplicate to ensure biological reproducibility.

### Statistical validation of the data

GraphPad Prism V8 software was employed for all statistical analyses and figure generation. A two-way analysis of variance (ANOVA) was performed to assess statistical significance between groups, followed by post-hoc multiple comparisons using Tukey’s test. The statistical threshold for significance was set as follows: *P*-values less than 0.0001 were denoted as ****, and *P*-values less than 0.0004 were denoted as ***.

## RESULTS

### Sample collection

All the water samples were collected from different effluents of Gomti river of Lucknow near the hospital waste, industrial waste, and domestic waste discharge. These regions are Ghaila: 26.91° N, 80.88° E; Indira Nahar: 26.83° N, 81.07° E; Mohanlalganj: 26.69° N, 80.99° E; BBAU: 26.76° N, 80.93° E; Telibagh: 26.79° N, 80.94° E; Sharda Nahar: 26.76° N, 81.83° E; Bakshi ka Talab: 26.98° N, 80.92° E; Rajanikhand: 26.78° N, 80.93° E; Lalbagh: 26.85° N, 80.94° E; Hasanganj: 26.87° N, 80.92° E.

### Elemental analysis

**Fig 1:**
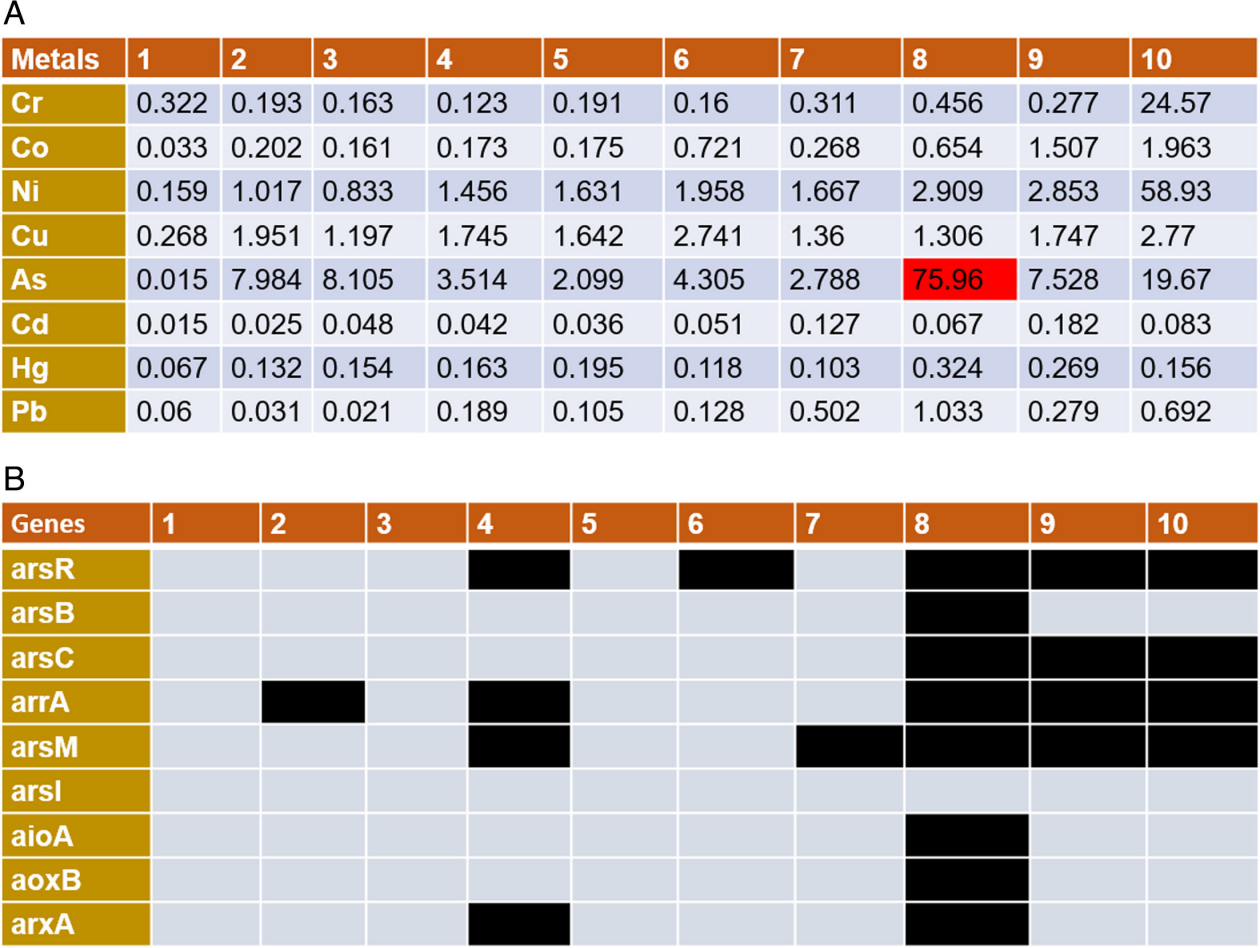
Elemental analysis of collected water samples and assessment of arsenic metabolizing gene presence. (**A**) The elemental analysis shows the concentration of various heavy metals in the collected water samples labeled 1 to 10. Among these, sample number 8 exhibits the highest concentration of arsenic, measured at 75 ppb, which is highlighted in the red box. (**B**) The presence of arsenic-metabolizing genes in the water samples is indicated by black boxes, while their absence is shown by gray boxes. Sample number 8 displays the maximum number of arsenic-metabolizing genes, with the exception of the demethylation gene *ars*I.

The concentration of heavy metals across the samples (from samples 1 to 10), as shown in Fig. 1A, ranged from 0 to 100 ppb. Chromium levels were detected between 0.1 and 24 ppb, with the highest concentration observed in sample 10. Cobalt was present in concentrations ranging from 0.03 to 1 ppb, while nickel concentrations varied between 0.1 and 58 ppb, with sample 10 also exhibiting the highest nickel concentration (58 ppb). Copper concentrations ranged from 0.2 to 2 ppb across the samples. As levels were detected between 0.01 and 75 ppb, with sample 8 showing the highest As concentration (75 ppb), exceeding the World Health Organization’s (WHO) maximum permissible limit of 10 ppb. Cadmium and mercury were found in trace amounts, ranging from 0.01 to 0.1 ppb. Notably, higher levels of chromium, nickel, copper, and As were detected in the Lucknow sample (sample 10) compared with other locations. However, since our study focused on As bioremediation, further analyses were conducted on sample 8, which had the highest As concentration (75 ppb).

### As-metabolizing genes

The assessment of genes indicated the presence of numerous As-metabolizing genes in the water samples. As shown in Fig. 1B, As-metabolizing genes were detected in all samples except for samples 1, 3, and 5. The genomic signatures of the As resistance system (*ars*RBC) were present in nearly all the water samples. Prokaryotic genomes, including accessory plasmids, transposons, and genomic islands, often harbor multiple *ars* operons with varying genes and configurations, providing resistance to inorganic As. In addition to these widely distributed *ars* gene clusters, recent studies have identified several genes that detoxify organic As derivatives in microbial cells, thereby expanding the range of As tolerance in microorganisms. Several genes responsible for arsenite oxidation (*arx*A, *aio*A, *aox*A), arsenate reduction (*arr*R), As methylation (*ars*M), and demethylation (*ars*I) were detected in water samples (Fig. 1B). No further analyses were performed on samples 1, 3, and 5 as they did not reveal the presence of any As-related genes. Sample 2 exhibited only the arsenate reductase gene (*arr*A), which reduces arsenate to arsenite. Sample 4 contained genes for arsenate reduction (*ars*R, *arr*A), arsenite methylation (*ars*M), and arsenite oxidation (*arx*A). Sample 6 contained only the arsenate reduction gene (*ars*R), while sample 7 exhibited only the arsenite methylation gene (*ars*M). Sample 8 contained a complete As resistance operon (*ars*R, *ars*B, and *ars*C) as well as genes for arsenite methylation, arsenite oxidation, and arsenate reduction, and hence for its volatilization. As a result, sample 8 was further selected for all subsequent studies. This study has further focused on identifying and assessing the activity of the As resistance gene cluster (*ars*RBC) and the As metabolism genes *arr*A, *aio*A, *aox*B, *arx*A, *ars*M, and *ars*I among the selected isolates for identification of new As-metabolizing genes.

### Isolation of microbial consortium

**Fig 2:**
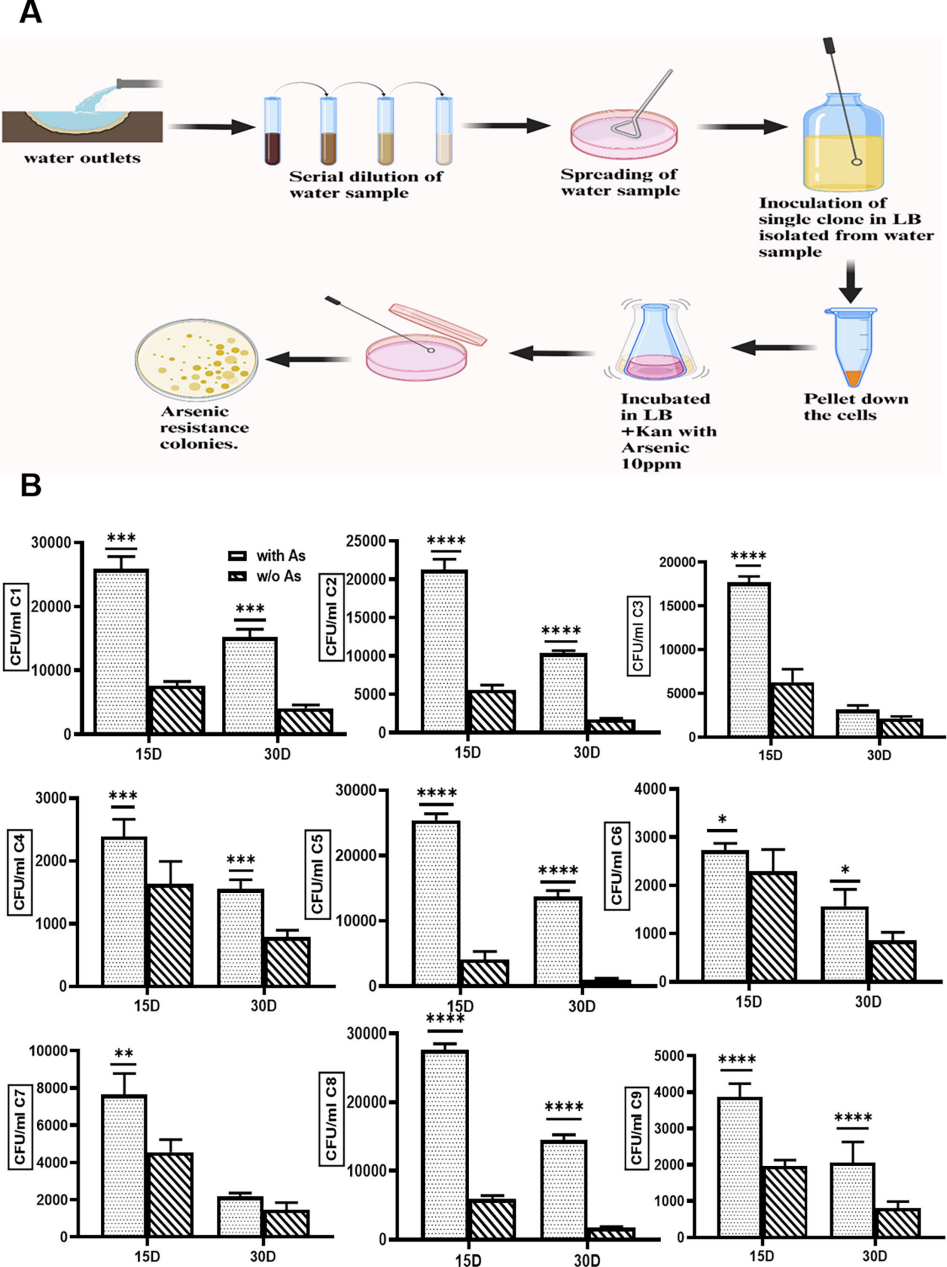
Arsenic-resistant bacteria isolation method and survival assessment in the presence of arsenic over 30 days. (**A**) The method used for isolating bacteria from water sample number 8 resulted in a total of nine colonies. (**B**) Survival of all isolated colonies, labeled C1 to C9, in the presence of 10 ppm arsenic, was assessed for up to 30 days. Data are expressed as means ± SD (*n* = 3). Asterisks (*) indicate significant differences among the CFU/mL after 15 and 30 days of incubation in the presence and absence of arsenic (P* < 0.0001).

Sample no. 8 contained 75 ppb of As, which exceeds the WHO recommended safe limit for As in water. In addition to the elevated As levels, this sample also exhibited multiple As-resistance genes like *ars*RBC, *arr*A, *aio*A, *aox*B, *arx*A, *ars*M, and *ars*I, many with putative As-metabolizing functions. In Fig. 2A, the collected water sample no. 8 is spread out on LB agar plates after being serially diluted. The cells were pelleted down and inoculated into LB + kanamycin with 10 ppm of As after incubation, which produced a single colony. Once the As and antibiotic-resistant microorganisms were isolated, this colony was further inoculated into M9 minimal media containing 10 ppm As and incubated for 30 days at 27°C.

After incubation, only nine colonies survived in the presence of As. Changes in colony growth were observed at 15 and 30 days post-inoculation, and colony-forming units per milliliter (CFU/mL) were determined (Fig. 2B). After 15 days of incubation, isolate C1 exhibited approximately 26,000 CFU/mL, which was reduced to 20,000 CFU/mL after 30 days. Isolate C2 had 20,000 CFU/mL at 15 days and 10,000 CFU/mL at 30 days. Isolate C3 started with 18,000 CFU/mL at 15 days, decreasing to 4,000 CFU/mL by 30 days. Isolate C4 showed 2,500 CFU/mL at 15 days, which was reduced to 1,500 CFU/mL after 30 days. Isolate C5 had 28,000 CFU/mL at 15 days and decreased to 18,000 CFU/mL after 30 days. Isolate C6 exhibited 2,500 CFU/mL after 15 days and 1,500 CFU/mL at 30 days. Isolate C7 showed 5,500 CFU/mL at 15 days and decreased to 2,000 CFU/mL after 30 days. Finally, isolate C8 had 28,000 CFU/mL at 15 days, which dropped significantly to 2,000 CFU/mL after 30 days. Isolate C9 had 4,000 CFU/mL at 15 days, and after 30 days, it became 2,000 CFU/mL (Fig. 2B). Among these, three isolates, i.e., C1, C5, and C8, were selected for further experiments due to their higher survival rates even after 30 days of incubation in the presence of As (Fig. 2B).

### Assessment of differential As removal by selected isolates

**Fig 3:**
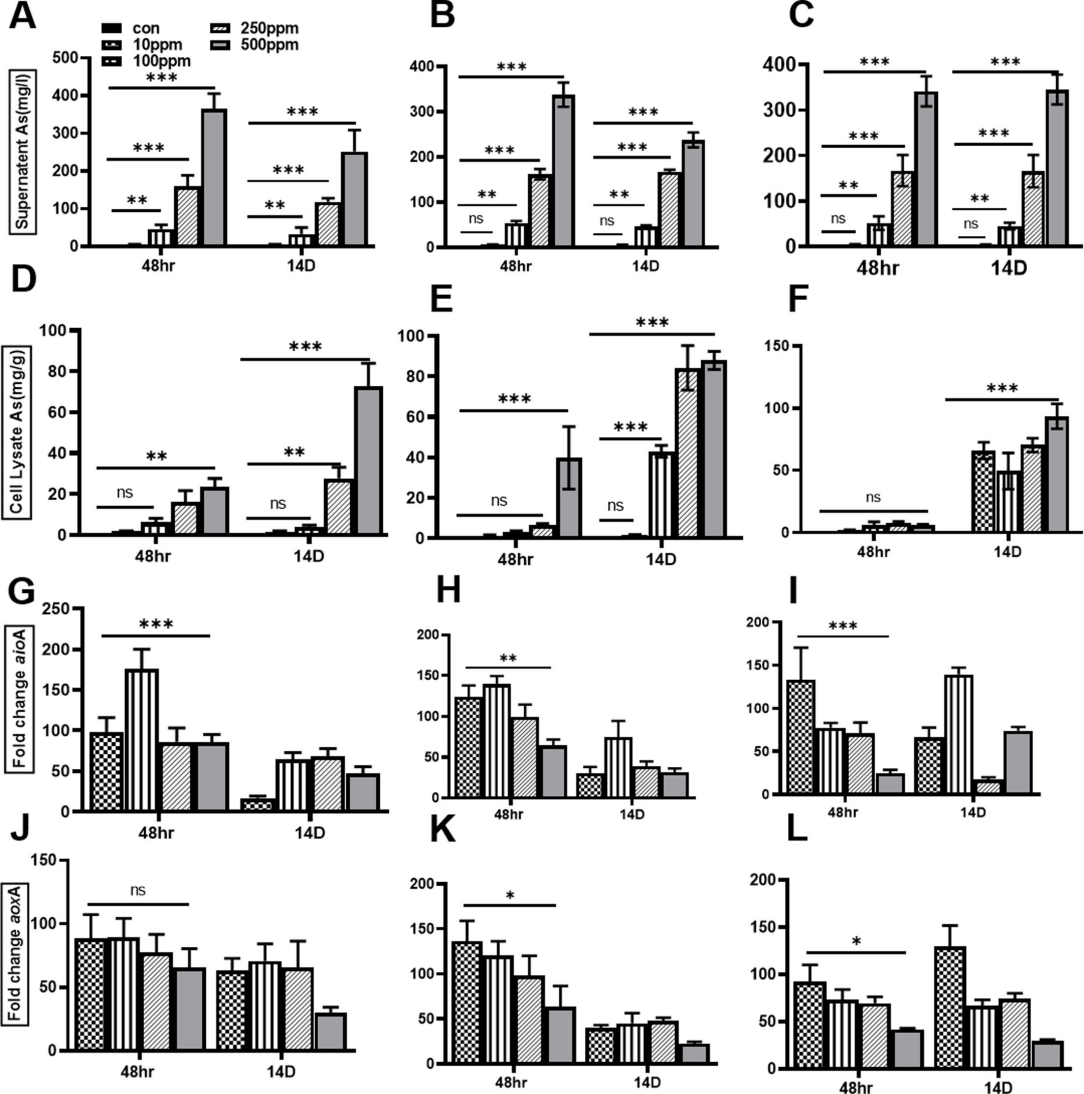
Assessment of arsenic in M9 culture medium/cells after 48 h and 14 days of incubation and quantification of arsenic oxidative genes (*aio*a, *aox*a). (**A**) Arsenic concentration in the supernatant after 48 h and 14 days of incubation with colony C1. (**B**) Arsenic concentration in the supernatant after 48 h and 14 days of incubation with colony C5. (**C**) Arsenic concentration in the supernatant after 48 h and 14 days of incubation with colony C8. (**D**) Arsenic accumulation by the cells after 48 h and 14 days of incubation with colony C1. (**E**) Arsenic accumulation by the cells after 48 h and 14 days of incubation with colony C5. (**F**) Arsenic accumulation by the cells after 48 h and 14 days of incubation with colony C8. (**G**) Real-time fold change expression of the *aio*A gene in Colony C1 after 48 h and 14 days of incubation. (**H**) Real-time fold change expression of the *aio*A gene in Colony C5 after 48 h and 14 days of incubation. (**I**) Real-time fold change expression of the *aio*A gene in Colony C8 after 48 h and 14 days of incubation. (**J**) Real-time fold change expression of the *aox*A gene in Colony C1 after 48 h and 14 days of incubation. (**K**) Real-time fold change expression of the *aox*A gene in Colony C5 after 48 h and 14 days of incubation. (**L**) Real-time fold change expression of the *aox*A gene in Colony C8 after 48 h and 14 days of incubation. Data are expressed as means ± SD (*n* = 3). Asterisks (*) represent significant differences among the CFU/mL after 15 and 30 days of incubation in the presence and absence of arsenic. Significance values are indicated as follows: **P < 0.0001, *P < 0.004, and *P* = 0.003. Control represents media only.

It was found that all three isolates C1, C5, and C8 could lower the amount of As in the growth medium by approximately 50% (Fig. 3A through C). The three isolates also showed different uptake of arsenic (As). Isolate C1 began As accumulation after 48 h, with concentrations ranging from 5 to 50 mg/g, as shown in Fig. 3D. This accumulation was markedly accelerated after 14 days of incubation. The As accumulation pattern for isolate C5 is shown in Fig. 3E, whereas the As accumulation in cells treated with isolate C8 is shown in Fig. 3F. After incubations, isolate C8 notably showed a minimal uptake of 50 mg/g of As. Both isolates C5 (Fig. 3E) and C8 (Fig. 3F) demonstrated strong resistance to As, enabling prolonged survival in its presence. As a result, C5 and C8 isolates were the focus of later experimental studies.

### Relative expression profiling of arsenite oxidation genes in selected isolates

Following 48 h and 14 days of incubation, cells of selected isolates were harvested, and RNA was extracted for qPCR analysis to assess the relative expression of genes involved in As metabolism. The qPCR results revealed an upregulation of the arsenite oxidase genes, *aio*A and *aox*B, across all isolates (Fig. 3G through L). However, each isolate exhibited distinct gene expression profiles at varying As concentrations. Isolate C1 (Fig. 3G and J) displayed a 100- to 200-fold increase in *aio*A and *aox*B expression after 48 h, which further increased to 20- to 60-fold after 14 days of incubation. Similarly, isolate C5 (Fig. 3H and K) showed a 100- to 180-fold increase in the expression of *aio*A and *aox*B after 48 h, with a subsequent 30- to 70-fold increase after 14 days. In isolate C8 (Fig. 3I and L), *aio*A and *aox*B expression increased 30- to 130-fold after 48 h, and this elevated expression level was sustained for up to 14 days.

### As volatilization by selected isolates at an environmentally relevant concentration

**Fig 4:**
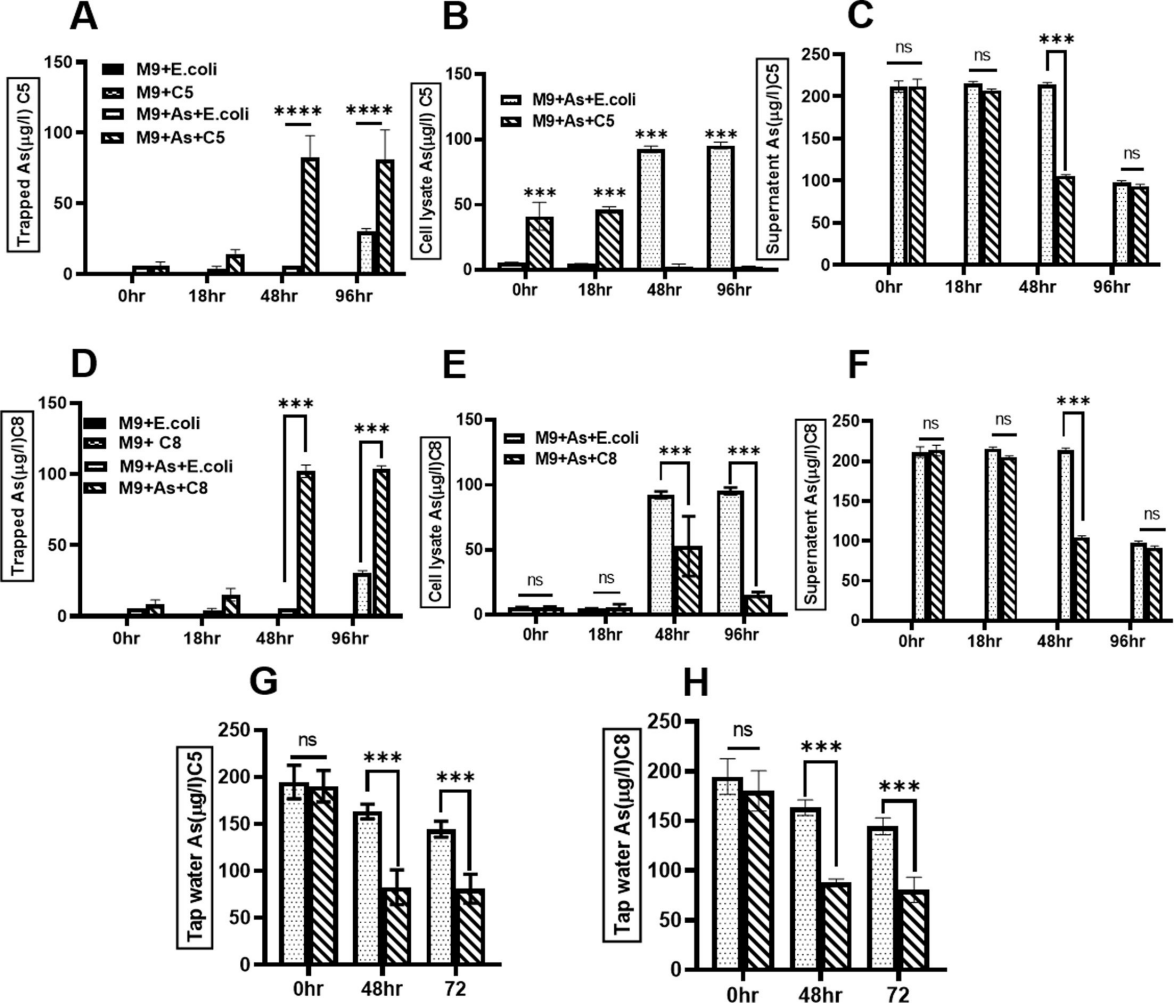
Evaluation of arsenic volatilization by isolated colonies C5 and C8 incubated in M9 media (A to F) and tap water (G and H) with arsenic. (**A**) Concentration of arsenic trapped in the nitrocellulose membrane, volatilized by Colony C5. (**B**) Arsenic concentration remained in the supernatant medium after 18, 48, and 96 h of incubation with colony C5. (**C**) Amount of arsenic accumulated by the cells when incubated with Colony C5 after 18, 48, and 96 h. (**D**) Concentration of arsenic trapped in the nitrocellulose membrane, volatilized by Colony C8. (**E**) Arsenic concentration remained in the supernatant medium after 18, 48, and 96 h of incubation with Colony C8. (**F**) Amount of arsenic accumulated by the cells when incubated with Colony C8 after 18, 48, and 96 h. (**G**) The concentration of arsenic remained in tap water after 48 and 72 h of incubation with colony C5. (**H**) The concentration of arsenic remained in tap water after 48 and 72 h of incubation with colony C8. Data are expressed as means ± SD (*n* = 3). Asterisks (*) indicate significant differences in CFU/mL after 15 and 30 days of incubation in the presence and absence of arsenic, with significant values as follows: **P < 0.0001, *P < 0.004, and *P* = 0.003.

The volatilized As, residual As in the supernatant, and As accumulated by the cells were quantified after 18, 48, and 96 h of incubation using atomic fluorescence spectrometry (AFS), as depicted in Fig. 4A through H. Figure 4A illustrates the concentration of trapped As by Colony C5 over 0, 18, 48, and 96 h of incubation. The concentration found in the nitrocellulose membrane was 1 ppm. Figure 4B shows the As concentration accumulated by C5 during the same time intervals. Isolate C5 accumulated As up to 18 h, whereas *E. coli*, used as the control, continued to absorb As after 18 h and up to 96 h, which resulted in decreased cell growth due to As toxicity. Figure 4C shows the remaining As concentration in the supernatant after 0, 18, 48, and 96 h of incubation with colony C5. Supernatant has 0.1 ppm of As after 48 and 96 h of incubation with colony C5, which is a 50% reduction of the given As concentration, i.e., 0.2 ppm, while *E. coli* As reduction after 96 h because of their accumulation. Figure 4D illustrates As volatilization by isolate C8, with approximately 50% of the supplied As (around 0.1 ppm) trapped in the membrane. This volatilization process was initiated after 18 h of incubation and persisted for 96 h. The control conditions employed here were identical to those used for isolate C5 in the previous experiment. The amount of As accumulated in the cell pellet after 18, 48, and 96 h of incubation is shown in Fig. 4E. Accumulation began in both isolate C8 and control *E. coli* cells after 18 h. However, while accumulation ceased in isolate C8 after 48 h, it continued in *E. coli* for up to 96 h. Figure 4F presents data on the As reduction in the media after 18, 48, and 96 h of incubation, revealing a 50% decrease in concentration compared with the initial As dose. Based on these observations, it has been concluded that isolates C5 and C8 volatilized a more significant amount of As than *E. coli*.

The isolates C5 and C8 were inoculated into regular tap water, with *E. coli* cells used as a control to validate these results further. Figure 4G depicts the As concentration in tap water after 48 and 72 h of incubation with isolate C5. After 48 h, As concentration was reduced by 50% compared with the control with *E. coli*, and this reduction continued through 72 h. Similarly, Fig. 4H shows the decrease in As concentration in tap water following incubation with isolate C8 for 48 and 72 h. Isolate C8 also achieved a 50% reduction in As compared with the *E. coli* control strain after incubations.

### Whole genome assembly, taxonomy, and annotation of selected isolates

The sequenced and assembled genome of the isolated strains was assessed for completeness and contamination. The consensus sequence of isolate C5 showed its lineage to the class Gamma-proteobacteria group, showing 100% completeness, 0.54% contamination, and no strain heterogeneity in the genome assembly. For isolate C8, with its lineage related to the family Xanthomonadaceae, the genome exhibited 0.00% contamination and 94.51% completeness with no strain heterogeneity, further confirming the purity of the genomes. The GTDB-based taxonomic validation of isolate C5 identified it as a bacterial microorganism of class Gamma-proteobacteria species *Providencia vermicola*, showing 99.34% identity with FAST-ANI reference *Providencia* GCF_010748935.1. Similarly, isolate C8 was identified as a bacterial isolate of class Gamma-proteobacteria species *Stenotrophomonas maltophilia* with a 95.7% identity with the FAST-ANI reference genome of *Stenotrophomonas maltophilia* GCA_014171555.1 (Table 3).

**TABLE 3:** GTDB taxonomy of the isolated strain

User genome	Classification	FastANI reference	FastANI	Closest placement ANI	Other related reference
2aAssembly	d_Bacteria;p_Proteobacteria;c_ Gammaproteobacteria;o_ Enterobacteriales;f_ Enterobacteriaceae;g_Providencia;s_ *Providencia vermicola*	GCF_010748935.1	99.34	99.34	GCF_900455155.1,s_Providencia stuart
3d1Assembly	d_Bacteria;p_Proteobacteria;c_ Gammaproteobacteria; o_Xanthomonadales;f_Xanthomonadaceae; g_Stenotrophomonas;s_ *Stenotrophomonas maltophilia*_AK	GCA_014171555.1	95.77	95.77	GCF_006970445.1;s_Stenotrophomonas

**Fig 5:**
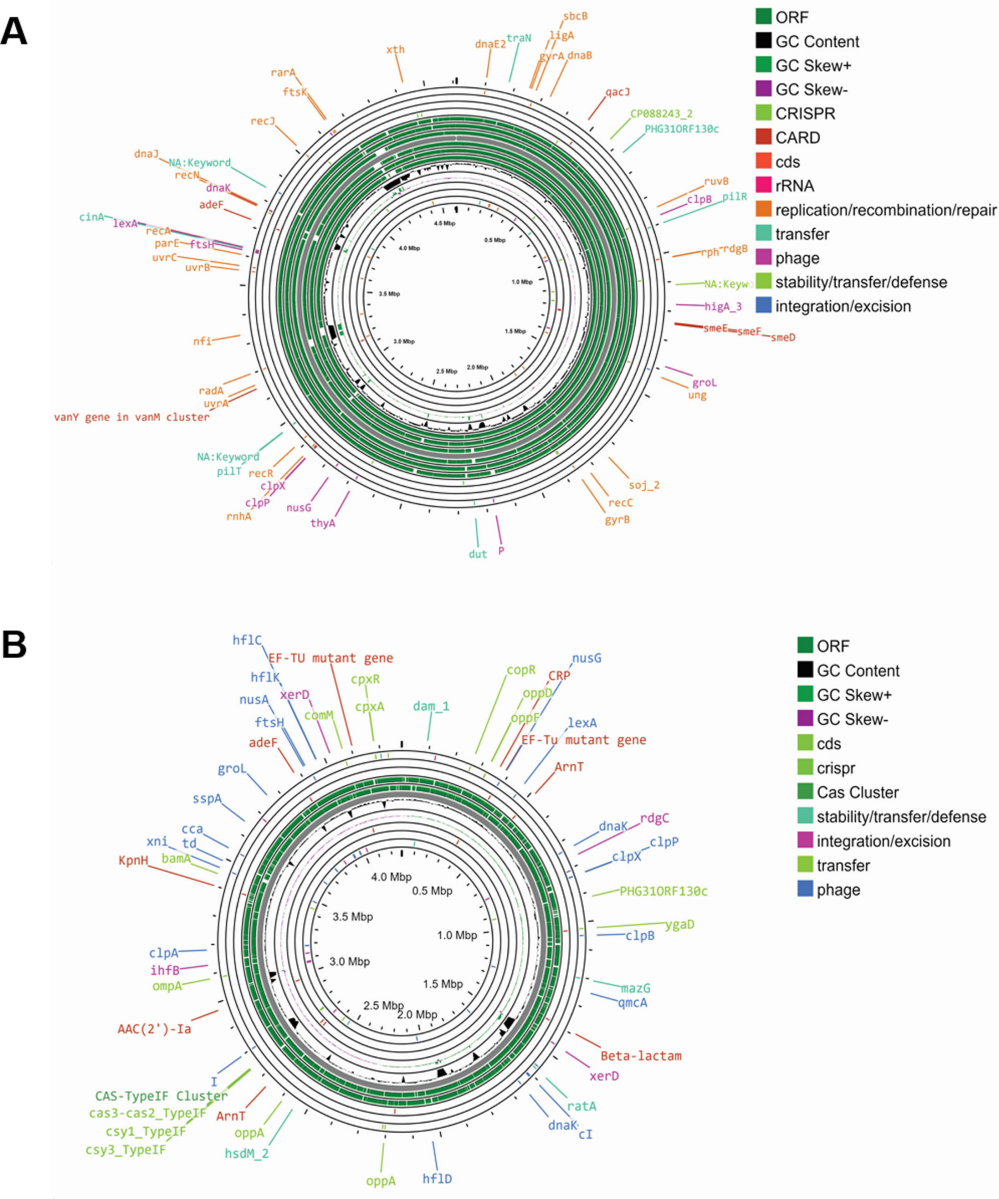
Genome map of the isolated colonies C5 and C8 showing the presence of ORFs, CRISPR, cas cluster, and other gene clusters. (**A**) Genome map of Colony C8 (*Stenotrophomonas*) displaying ORFs, GC content, CRISPR, CARD, rRNA, genes related to replication/recombination/repair/transfer/phage, and genes involved in stability/transfer/defense, as well as integration/excision genes. (**B**) Genome map of the isolated Colony C5 (*Providencia*) showing ORFs, GC content, GC skew, CRISPR, Cas Cluster, genes involved in stability/transfer/defense, integration/excision, transfer, and phage.

The genome map of isolates C5 and C8 (Fig. 5A and B) reveals a high GC content, numerous open reading frame (ORF) regions, and the presence of genetic elements related to the Clustered Regularly Interspaced Short Palindromic Repeats (CRISPR), resistance against different antimicrobials, including antibiotics and heavy metals, and phage regions were identified in genome assemblies after annotation. Coding sequences (CDS) for functions related to rRNA, replication, recombination, repair, transfer, stability, and defense were also identified. Antimicrobial resistome profiling of selected isolates revealed the presence of genes conferring defense against different antibiotics, heavy metals, and various other stressors. The highest abundance was found for the genes conferring resistance against antibiotics (*mde*A, *ade*L, *blt*D, *mtr*F, *rob*A, *vca*M, *acr*B, *ade*D, *acr*R, *cme*R, *ebr*B, *emr*A, *emr*B, *emr*D, and *emr*D-3), heavy metals, especially copper, zinc, nickel, etc. (*cor*R, *mod*C, *fet*A, *fet*B, *hmr*R, *mnt*H, *mnt*P, *rcn*R, *teh*B, *yfe*A, *yfe*B, *yfe*C, and *yfe*D), and other stress responses (*cpx*R, *bhs*A, *ibp*A, *nlp*E, *yod*A, and *zra*S).

### Arsenic volatilization genes

**Fig 6:**
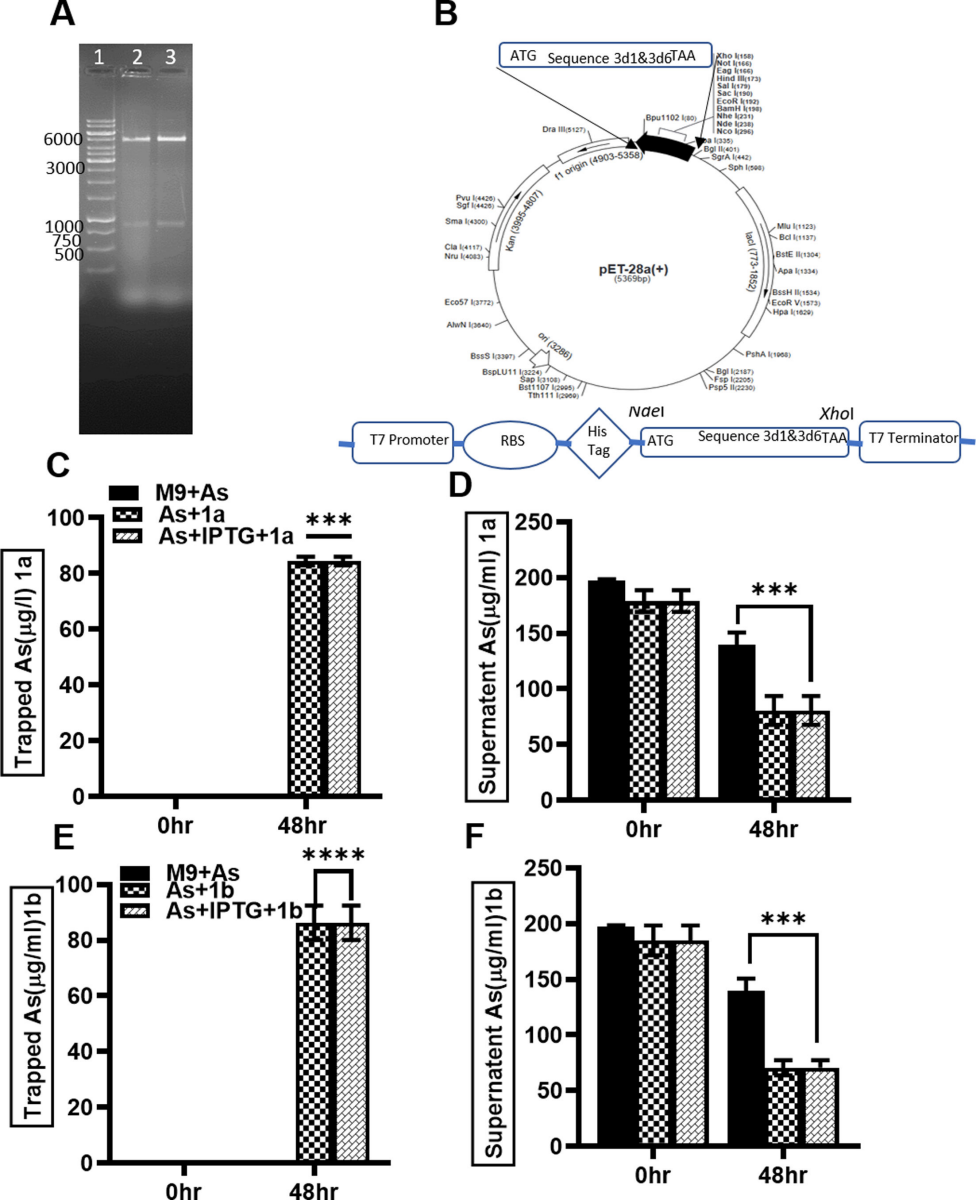
Cloning of isolated methyltransferase sequence into *E. coli* and validation of its volatilization ability. (**A**) Isolated sequences on agarose gel: Lane 1 shows the 1 Kb DNA ruler, Lane 2 shows Sequence 3d1, Lane 3 shows Sequence 3d6. (**B**) Schematic diagram of the pET 28(+)a vector with the insertion site of the sequences highlighted by a bold black arrow. (**C**) Arsenic concentration volatilized by construct 1a containing Sequence 1, with media containing only arsenic used as a control. (**D**) Arsenic reduction by construct 1a containing Sequence 1, with media containing only arsenic used as a control. (**E**) Arsenic concentration volatilized by construct 1b containing Sequence 8. (**F**) Arsenic reduction by construct 1b containing Sequence 8. Data are expressed as means ± SD (*n* = 3). Asterisks (*) indicate significant differences in the CFU/mL after 15 and 30 days of incubation in the presence and absence of arsenic, with significant values as follows: **P < 0.0001.

Genome annotations of C5 and C8 isolates revealed several heavy metal resistance genes and 20 potential SAM-dependent methyltransferase genes. The expression of the genes was then checked using RT-PCR. Among 20 putative genes, only two methyltransferase genes were observed to be expressed in isolate C8 of *Stenotrophomonas maltophilia,* as confirmed with RT-PCR profiling. However, none of such genes expressed in the C5 isolate of *Providencia* sp. complete sequences of expressed genes were mined and further subjected to *in silico* validation. BLASTx of two putative sequences (3d1 and 3d6) against the non-redundant database of NCBI identified them as SAM-dependent methyltransferases of *Stenotrophomonas* sp. origin having 100% identities with reference genomes. Conserved domains of S-adenosylmethionine-dependent methyltransferases (SAM or AdoMet-MTase) proteins were also predicted in the putative peptides, confirming their function as methyltransferases. The PCR amplification products with gene-specific primers and genomic DNA of *Stenotrophomonas maltophilia* C8 as a template resulted in a band of ~1 kbp on agarose gel, which is equivalent to the predicted gene size, confirming the quality of genome assembly and annotation (Fig. 6A). The genes were cloned in the pET28a (+) vector expression vector to express the recombinant protein with an N-terminal 6X histidine tag (Fig. 6B).

### As volatilization in recombinant host

After 48 h of incubation, transformed *E. coli* cells containing the plasmid carrying sequences 3d1 and 3d6 mentioned in Table 2. Constructs Seq1 and Seq2 were analyzed analyzed for As volatilization. These constructs are denoted as 1a for having sequence 3d1 and 1b for having sequence 3d6. The extent of As volatilization by the transformed construct 1a is illustrated in Fig. 6C, where M9+As represents minimal M9 media with As only, while “As+1a” refers to M9 media with construct 1a and As, and “As+1a + IPTG” is the M9 media containing IPTG for induction and containing construct with Sequence 3d1. The cells harboring plasmid with Seq3d1 showed As volatilization in both cases, with and without the IPTG induction. The aforesaid cells reduced the 60% concentration of As from the media after 48 and 72 h of incubation (Fig. 6D). Similarly, cells containing sequence 3d6 denoted as 1b could volatilize 50% of As from the media, both in the presence and absence of IPTG, after a similar duration of incubation (Fig. 6E). About a 50% reduction of arsenic by these cells has been observed after 48 and 72 h of incubation (Fig. 6F).

## DISCUSSION

As is a naturally occurring element found in the Earth’s crust, which is released into the environment through natural processes like volcanic activity and weathering of rocks, as well as by human activities such as mining, smelting, and using As-containing pesticides. As contamination is a significant environmental and public health issue due to its toxicity and long persistence. Primary sources of As include groundwater, soil accumulation, air pollution from industrial processes, and burning of fossil fuels. Long-term exposure to As-contaminated water can lead to severe health issues, including various cancers, skin lesions, cardiovascular diseases, and developmental effects during pregnancy. As exists in different forms, with inorganic As being highly toxic.

Microorganisms play a vital role in As detoxification through mechanisms like arsenite efflux, arsenate reduction, and As methylation. Several microbes play crucial roles in As detoxification through various biochemical pathways. These include *Pseudomonas*, commonly found in soil and water, known for its ability to oxidize arsenite (As(III)) to less toxic arsenate (As(V)), *Alcaligenes* and *Agrobacterium*, typically found in soil, also contribute to As oxidation, and *Herminiimonas arsenicoxydans*, known for colonizing As-rich environments, perform both As oxidation and efflux. Thermus species in hot springs oxidize arsenite, while *Achromobacter* and *Paraburkholderia* contribute through oxidation and detoxification pathways in various environments. *Comamonas* and *Klebsiella*, present in soil and water, are also involved in As detoxification. These microbes utilize oxidation, reduction, methylation, and efflux pathways to detoxify As, reducing its harmful environmental effects. Leveraging their natural detoxification capabilities to reduce As contamination can be an effective bioremediation strategy.

The present work focused on identifying the As-metabolizing bacteria from groundwater heavily contaminated with various heavy metals, including As. We then determined their potential for transformation and mobilization of As, with the aim of identifying the potential As volatilization genes. Volatilization can help in the permanent removal of As from groundwater, which is in contrast to other processes that only convert As to a less toxic form.

In Fig. 1, we report the levels of various heavy metals in the water samples collected from different contaminated water bodies. The presence of multiple metals was observed, and water sample number 8 showed the highest level of As along with other metals. Further, analysis for As-metabolizing genes also confirmed the presence of complete As resistance operons, including *ars*R, *ars*B, and *ars*C, along with genes involved in As methylation, volatilization, oxidation, and reduction (Fig. 1) in sample number 8. We, therefore, used this water sample for further selection and identification of As-metabolizing genes.

Further studies on the survival of bacteria in the presence of high As concentration were done in M9 minimal media (Fig. 2). Few bacteria from the selected water sample were found to survive up to 30 days in minimal media at 10 ppm As, suggesting the presence of unique genes and pathways in them. We further tested these bacterial clones for their As removal potential at high As concentrations (Fig. 3). This led to the selection of three clones (C1, C5, and C8), which could survive and reduce As in media by up to 50% even at As concentrations as high as 500 ppm within 14 days. In nature, microbial resistance to different As species at high concentrations has been previously observed. Resistance to doses above 10 mM As^3+^ and 100 mM As^5+^ is regarded as extremely high, but resistance to 30 mM As^3+^ or 300–500 mM As^5+^ is indicative of hyper-tolerance. Arsenate-resistant bacteria isolated from woodland soil (41) were found to grow up to concentrations of 250 mM of arsenate. A total of 64 As-resistant bacteria were isolated from the groundwater sample collected from As endemic areas in West Bengal, India, and were found to have higher tolerance to As3+ (maximum tolerable concentration [MTC]: C10 mM), As5+ (maximum tolerable concentration, MTC: C100 mM), and other heavy metals like Cu2+, Cr2+, Ni2+, etc. (MTC: C10 mM). They were also frequently found to have siderophore and arsenate reductase (49).

Using a specific setup for capturing the volatilized form of arsenic, we were able to accurately quantify the amount of As absorbed by bacteria, As remaining in the media, and the As volatilized out of the media (Fig. 4). When incubated with 0.2 ppm As, we found two clones, C5 and C8, to remove up to 50% of arsenic by volatilization within 48 h. In India, 0.2 ppm is the median As concentration reported in several As-contaminated sites.

Previous studies have also tried strategies to remove As by volatilization but with moderate success. As volatilization in flooded paddy soil with the addition of biochar (BC) and Fe–Mn–La-modified BC composites (FMLBCs) was studied previously (33). The amount of arsenic in medium-contaminated soil is reduced by 18% to 31% and in highly contaminated soil by 15% to 25% using this biochar. Soil enzyme activity increased, and the relative abundances of Proteobacteria and Actinobacteria changed with the addition of FMLBCs. Chen et al. (30) findings suggested that a combination of bioaugmentation with *P. putida* and biostimulation with RS/BC + RS is a potential strategy for bioremediation of arsenic-contaminated soils by enhancing As methylation and volatilization under non-flooded conditions.

Guarino et al. (31) assayed biophytoremediation technology using *Arundo donax* L., assisted by plant growth-promoting bacteria (PGPB) consortium (BC) constituted of two strains of *Stenotrophomonas maltophilia* sp. and one of *Agrobacterium* sp., and also checked for their epigenetic response to As pollution. This technology removes more than 50% of the As from the soil, and the strain was tolerant to 185 ppm of As concentration. While in our case, we isolated a strain that is tolerant to 500 ppm of As and removing more than 50% of As from water solely.

Plant growth-promoting (PGP) traits, such as arsenite methyltransferase (*Pseudomonas oleovorans,* B4.10), arsenate reductase (*Sphingobacterium puteale*, B4.22), and arsenite oxidase (*Citrobacter sp*., B5.12), contribute to arsenic detoxification in contaminated soils, thereby enhancing plant survival and biomass. Since arsenic methyltransferase was inoculated, the As content of the rice grain was effectively reduced by 61%.

During the mesophilic and early thermophilic stages, the addition of biochar increases the amount of monomethyl arsenic acid and dimethylarsenic acid. It also facilitates arsenic volatilization during the maturing phase (60–80 days). By encouraging As methylation and volatilization through *Ensifer* and *Sphingobium* carrying arsC genes, as well as *Rhodopseudomonas* and *Pseudomonas* carrying arsM genes, the biochar amendment had an impact on the microbial communities.

Whole genome sequencing of Clones C5 and C8, which had high As volatilization ability, led to the identification of unique strains of *Providencia* and *Stenotrophomonas,* respectively (Fig. 5). *Providencia* sp. and *Stenotrophomonas* were discovered by Mustapha et al. (32) in the patient sample from a hospital with a nosocomial infection. Beekele et al. (34) identified and isolated bacteria that break down diesel from hydrocarbon-contaminated sites, such as Flower Farms and Soda Lakes. Six diesel degraders were subsequently found to be associated with the bacteria *Pseudomonas*, *Providencia*, *Roseomonas*, *Stenotrophomonas*, *Achromobacter*, and *Bacillus*. This work showed that it is possible to optimize bacterial species that have been isolated from hydrocarbon-contaminated and/or uncontaminated environments to use them as potential bioremediation agents for the removal of diesel. Sequencing also revealed the presence of metal resistance genes and genes involved in As methylation. We identified 20 putative SAM-dependent methyltransferases and checked their expression by real-time PCR analysis (Fig. 6). Two sequences, which showed a high level of expression by real-time PCR, were further isolated, cloned in a pET28a vector, and transformed in *E. coli*. The transformed *E. coli* were cultured in M9 media spiked with As. Upon IPTG induction, we observed a significant reduction in As from media, confirming the ability of the transformed clones to rapidly remove arsenic.

**Fig 7:**
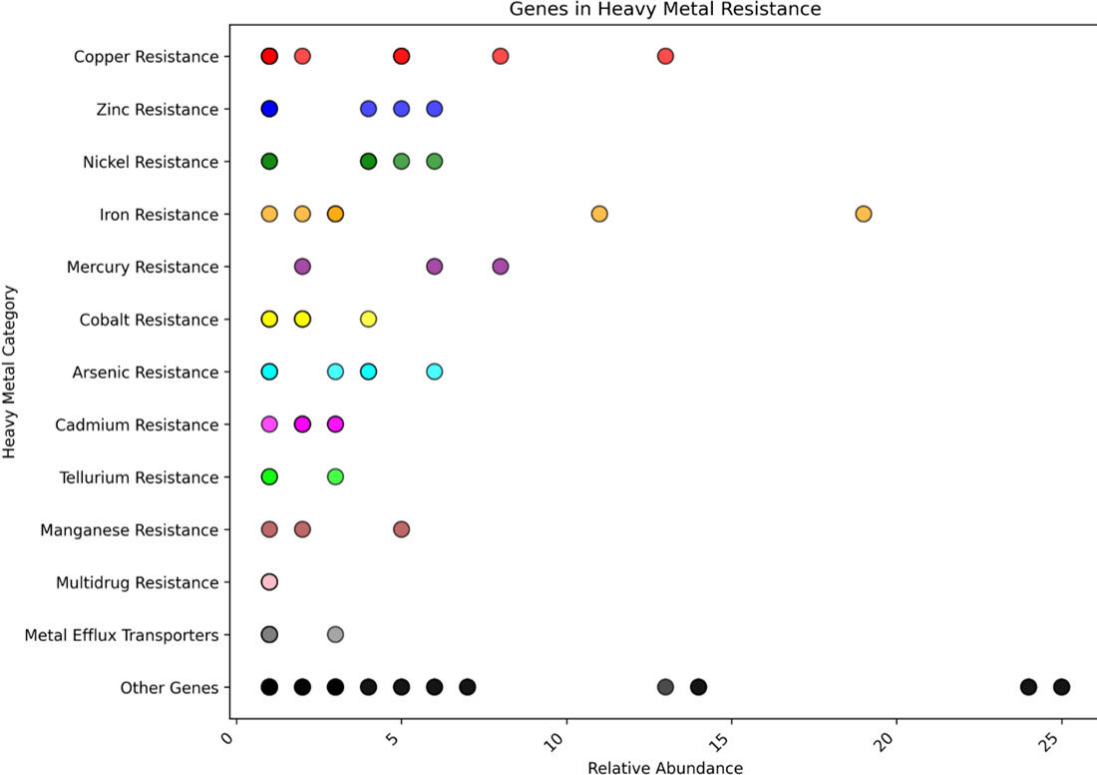
Resistome profile of the isolated colony C8. The figure shows the number of resistance genes that were found in the genome of the *Stenotrophomonas maltophilia* strain or C8 colony. They show the presence of the copper resistance gene, zinc resistance gene, nickel resistance gene, iron resistance gene, tellurium resistance gene, mercury resistance gene, cobalt resistance gene, arsenic resistance gene, manganese resistance gene, metal efflux transporter, and multidrug resistance gene.

**Fig 8:**
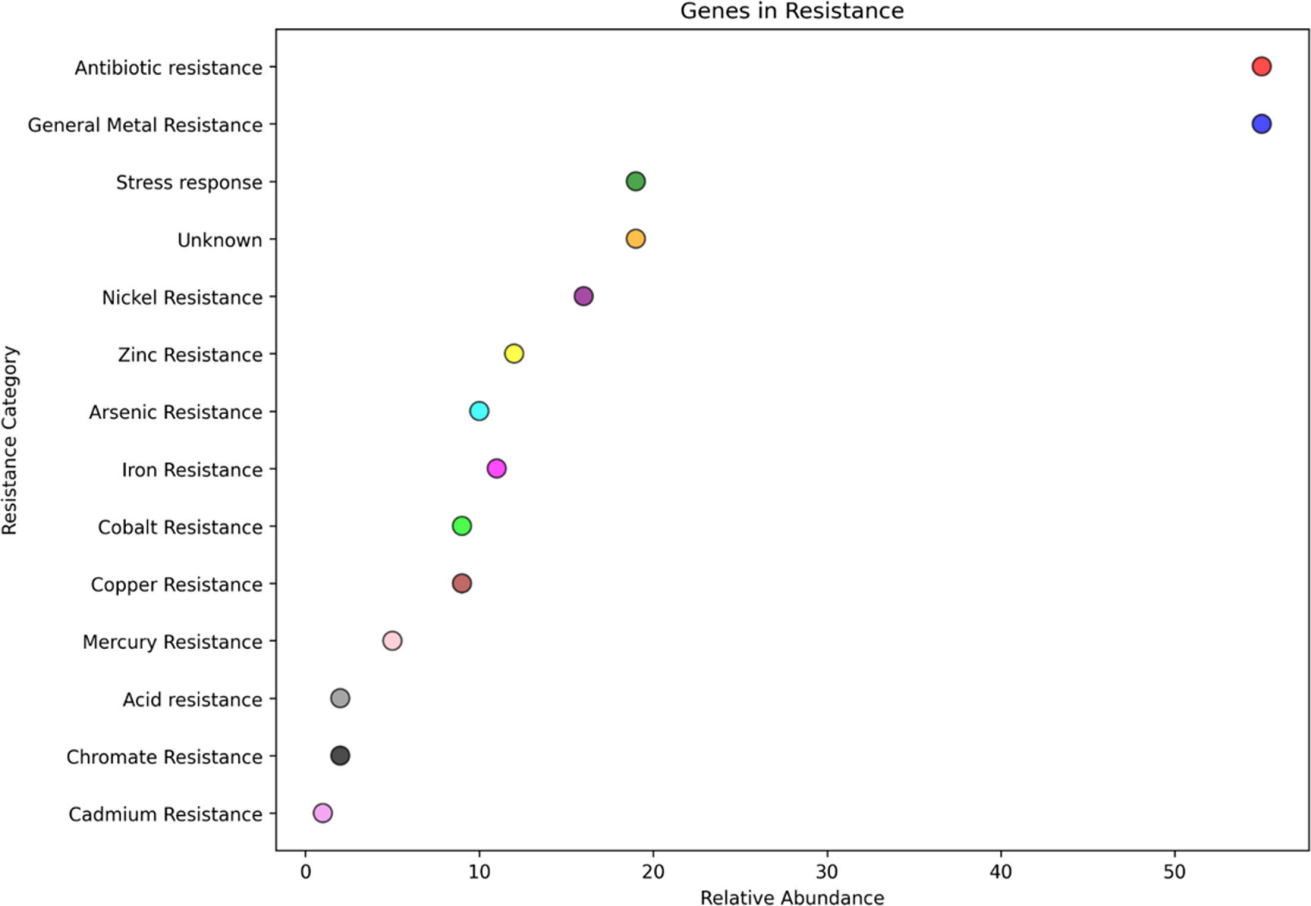
Resistome profile of the isolated colony C5. In this strain of *Providencia*, antibiotic resistance, general metal resistance, stress response, nickel resistance, arsenic resistance, zinc resistance, iron resistance, cobalt resistance, copper resistance, mercury resistance, acid resistance, chromate, and cadmium resistance genes are present in its genome.

Figure 7 represents the resistome profile of isolated clone C8, which has various heavy metal resistance genes like copper, zinc, nickel, iron, mercury, cobalt, arsenic, cadmium, tellurium, manganese, metal efflux transporter and also multidrug resistance gene. Figure 8 shows the presence of various heavy metal resistance genes in the resistome profile of isolated clone C5. This profile has nickel, arsenic, zinc, iron, cobalt, copper, mercury, resistance genes and also has antibiotic resistance, stress response, acid resistance and chromate resistance genes. Both of these resistome profiles show that both the isolated clone C5 and C8 have the capability to survive in the presence of heavy metal as well as in other stress conditions.

Along with genes like *arr*A, *aio*A, *aox*B, and *arx*A for arsenite oxidation and arsenate reduction via aerobic and anaerobic pathways, microorganisms also possess *ars*M and *ars*I genes involved in As methylation and demethylation, transforming toxic inorganic As into less toxic organic forms, which are more easily removed from the environment. Bacterial and fungal species, including *Penicillium* and *Aspergillus*, can have As methylation and volatilization, representing a crucial bioremediation strategy (50). Studies demonstrate that microbial As methylation significantly contributes to detoxification. For instance, *Stenotrophomonas maltophilia* strain SCSIOOM, isolated from the fish gut, unregulated *ars*H and *ars*RBC genes, enhancing As resistance and efflux ((51)). This microbial activity influences the biogeochemical cycle of As, playing a key role in As migration and transformation, especially in aerobic environments. Further research into microorganisms like *Stenotrophomonas maltophilia* has shown their capability for As methylation, reducing bioavailability and toxicity. For instance, a strain from paddy soil containing an arsenite methyltransferase gene (*ars*M) exhibited significant As methylation and volatilization when expressed in *E. coli* (23). This process underscores the potential of microbial bioremediation for As-contaminated environments. Additionally, studies have highlighted the effectiveness of biochar in enhancing As methylation and volatilization during composting. Biochar amendments increase the production of monomethyl As acid and dimethylarsinic acid during composting stages and promote As volatilization through bacteria carrying *ars*C and *ars*M genes (52).

Our studies through culture and whole genome sequencing of As-resistant strains have identified new As methyltransferase genes, providing a stable and effective mechanism for As detoxification. Expressing these genes in *E. coli* demonstrated rapid conversion of arsenite to volatile, methylated As (53). As contamination is a significant environmental and public health issue, affecting millions of people worldwide, microbial bioremediation can be used to clean up As-contaminated sites in a cheaper and sustainable way. Our study involved the identification and use of yet unknown As volatilization genes, and no genetic manipulation was done to alter their activity, which can facilitate their rapid transition to field conditions.

In conclusion, microbial As methylation and volatilization represent promising strategies for bioremediation of As-contaminated environments. The potential of microbial genes in these processes offers a path forward for developing effective and sustainable remediation technologies. Understanding microbial As metabolism and its genetic basis will continue to enhance our capabilities in environmental remediation.

### Conclusion

This study demonstrates the remarkable capabilities of the newly isolated *Stenotrophomonas maltophilia* strain in As volatilization and survival under extreme conditions. This autotrophic strain successfully volatilized As from water in less than 48 h and survived for up to 72 h in As-spiked water. Notably, the strain could persist for up to 30 days without any carbon sources, even in As concentrations as high as 500 ppm. Whole genome sequencing of the strain revealed a robust genetic arsenal, including multiple heavy metal resistance genes for copper, zinc, nickel, mercury, cobalt, cadmium, tellurium, manganese, and multidrug resistance genes. Notably, the genome also identified potential As methyltransferase genes critical for As biotransformation and detoxification. These genes could play a pivotal role in As methylation and volatilization, offering an eco-friendly solution to As-contaminated water. Given its high tolerance and efficient As removal capabilities, this strain holds excellent potential for future bioremediation applications, particularly for detoxifying As-contaminated water sources. Furthermore, the strain’s ability to volatilize As without external carbon sources highlights its adaptability and suitability for large-scale environmental applications, including immobilized bioreactors and *in situ* water treatment systems.

## Data Availability

The high-quality fastq reads, as well as the assembled genome of *Stenotrophomonas* sp. 3diitr2024, have been submitted to DDBJ/ENA/GenBank under accession number JBHHMJ000000000.
